# Supplementary material for: Metabolic Response in Endothelial Cells to Catecholamine Stimulation Associated with Increased Vascular Permeability
Source: Int J Mol Sci. 2022 Mar 15;23(6):3162. doi: 10.3390/ijms23063162 (PMC8950318; doi:10.3390/ijms23063162)
Supplement: Supplementary file 1 [file ijms-23-03162-s001.zip › ijms-1623116-supplementary.pdf]

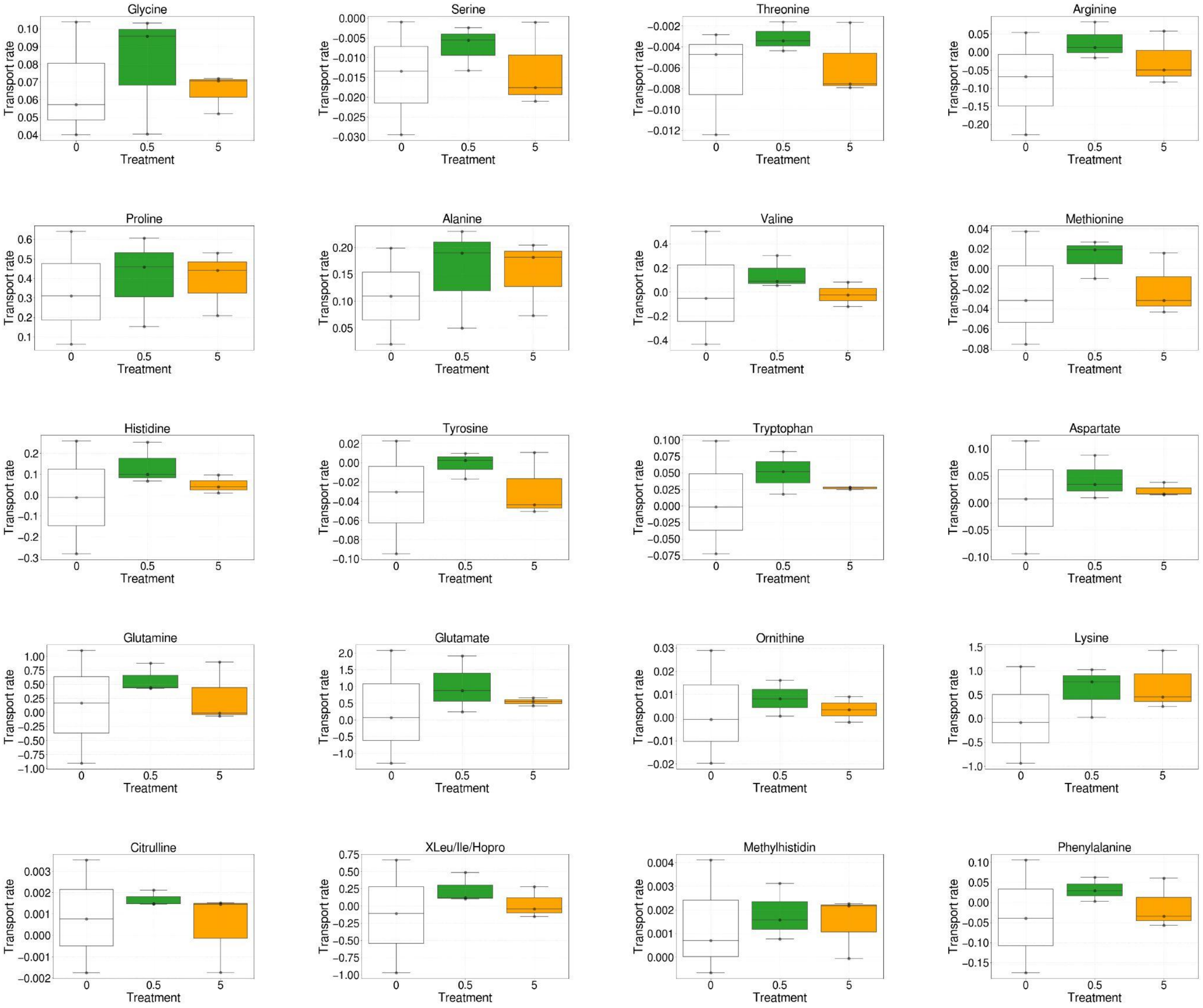

Supplementary Information Figure SIF1 | Effect of adrenaline-noradrenaline treatment on amino acid transport rates.

Positive rates indicate secretion; negative rates indicate uptake. Transport rate units are μM × 10^3 $cells^{-1}$ × day^{-1}. We found no particular trend in amino acid transport rates associated with treatment (0.5 and 5 μM of equimolar adrenaline-noradrenaline mix).

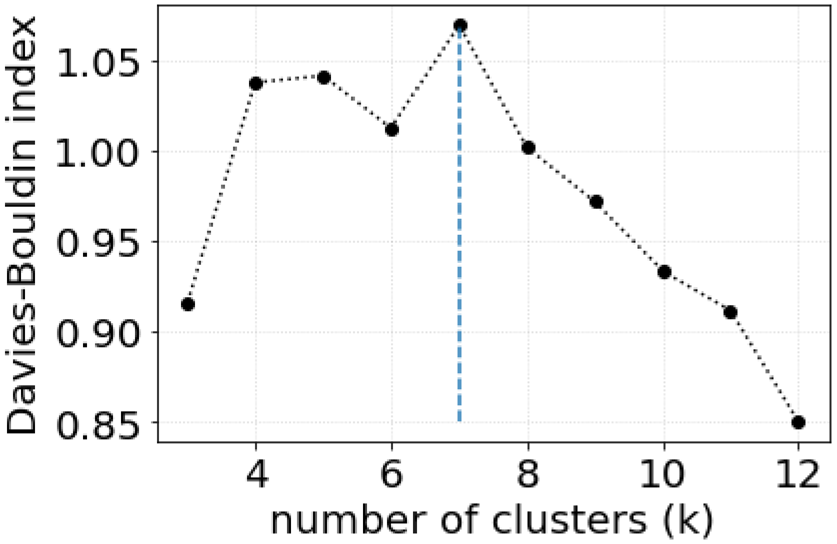

Supplementary Information Figure SIF2 | Quantitative identification of the optimal clustering partition.
We used the Davies-Bouldin index to quantitatively assess the optimal number of clusters. Blue dashed line marks the maximum value found, indicating that our dose-responding metabolic features are best grouped together into seven clusters.
